# Overexpression of zinc finger protein 384 (ZNF 384), a poor prognostic predictor, promotes cell growth by upregulating the expression of Cyclin D1 in Hepatocellular carcinoma

**DOI:** 10.1038/s41419-019-1681-3

**Published:** 2019-06-05

**Authors:** Lifeng He, Xiaoxiao Fan, Yirun Li, Mingming Chen, Bin Cui, Guoqiao Chen, Yili Dai, Daizhan Zhou, Xiaotong Hu, Hui Lin

**Affiliations:** 1 Department of General Surgery, Sir Run Run Shaw Hospital, School of Medicine, Zhejiang University, Hangzhou, China; 2 Biomedical Research Center, Sir Run Run Shaw Hospital, School of Medicine, Zhejiang University, Hangzhou, China; 3 School of Life Science and Technology, Nanyang Normal University, Nanyang, China; 4 Key Laboratory of Arrhythmias of the Ministry of Education of China, East Hospital, Tongji University School of Medicine, Institute of Medical Genetics, Tongji University, Shanghai, China

**Keywords:** Liver cancer, Liver cancer, Oncogenes, Oncogenes, Cell growth

## Abstract

Hepatocellular carcinoma (HCC) is a highly heterogeneous, multigene-driven malignant tumor. ZNF384 is an overexpressed gene with a high frequency of alteration in HCC, but research on the function of ZNF384 in HCC is lacking. In this study, the expression level of ZNF384 in HCC was analyzed through immunohistochemical (IHC) staining, Western blot analysis and qRT-PCR. We also generated ZNF384 knockdown and knockout HCC cell lines using short hairpin RNA (shRNA) and CRISPR/Cas9 systems. MTS, colony formation, and 5-ethynyl-20-deoxyuridine (EdU) assays; flow cytometry; and a xenograft mouse model were used to evaluate the effects of ZNF384 on cell proliferation. Western blot analysis, a dual luciferase reporter assay and a ChIP assay were performed to explore the potential mechanism. We found that overexpression of ZNF384 in HCC and elevated expression of ZNF384 in HCC tissues was significantly correlated with tumor recurrence (*P* = 0.0097). Kaplan–Meier survival analysis revealed that high expression levels of ZNF384 were correlated with poor overall survival (*P* = 0.0386). Downregulation of ZNF384 expression suppressed HCC cell proliferation by inhibiting the expression of Cyclin D1. These findings suggest that ZNF384 tends to act as an oncogene in the development of HCC. ZNF384 promotes the proliferation of HCC cells by directly upregulating the expression of Cyclin D1 and might serve as a prognostic predictive factor for HCC patients.

## Introduction

Hepatocellular carcinoma (HCC) is a heterogeneous disease with a poor 5-year survival rate of less than 10%^1^. Multiple risk factors involve in the initiation and progression of HCC including HBV and/or HCV infections, chronic alcohol consumption, metabolic syndrome, obesity and diabetes which contribute to the complex and extraordinarily heterogeneous characteristics of HCC^2^. In the era of advocating individualized treatment of cancers, the tumor heterogeneity greatly impedes the development of HCC molecular targeted drugs^3,4^. Genomic studies about HCC have depicted a map of molecular alterations in HCC. Regrettably, the targetable driven genes for treatment and the biomarkers for response of the targeted treatment is still mysterious^5,6^. As a result, the efficacy of targeted therapy including the sorafenib and regorafenib for the treatment of HCC is not satisfactory, especially in Chinese population^7,8^. It pushed us to explore the role of genes that have changed in HCC sequencing research in the development of HCC.

ZNF384 gene encodes a C2H2-type zinc finger protein which functions as a transcription factor regulates the transcription of the extracellular matrix genes^9^. The previous studies reported that ZNF384 fused with the TET family genes, including Ewing sarcoma breakpoint region 1 (EWSR1) gene, TATA box binding protein-associated factor (TAF15) and transcription factor 3 (TCF3) and played an important role in ALL^10,11^. Sakuma et al.^12^ found that the over expression of ZNF384 would promote the migration of melanoma cells. Mori et al.^13^ demonstrated that ZNF384 bound to APOBEC3B (A3B) promoter and functions as a modulator of A3B expression in cervical cancer. In addition, The Cancer Genome Atlas (TCGA) and Oncomine databases suggest that ZNF384 is an overexpressed gene with high frequency alterations in HCC. Although various evidences indicated that ZNF384 might be a potential oncogene, which promotes the occurrence and development of cancers, the research about ZNF384 in HCC is vacant.

In this study, we first identified the role of ZNF384 in the initiation and progression of HCC. We confirmed the overexpression of ZNF384 in HCC tissues. In addition, we characterized the oncogenes role of ZNF384 in HCC and explored the potential mechanism of ZNF384 in regulating cell proliferation in vivo and in vitro. Our results showed that ZNF384 promote tumor growth and may be a novel prognostic marker in HCC.

## Results

### Overexpression of ZNF384 in HCC tissues and cell lines

**Fig. 1:**
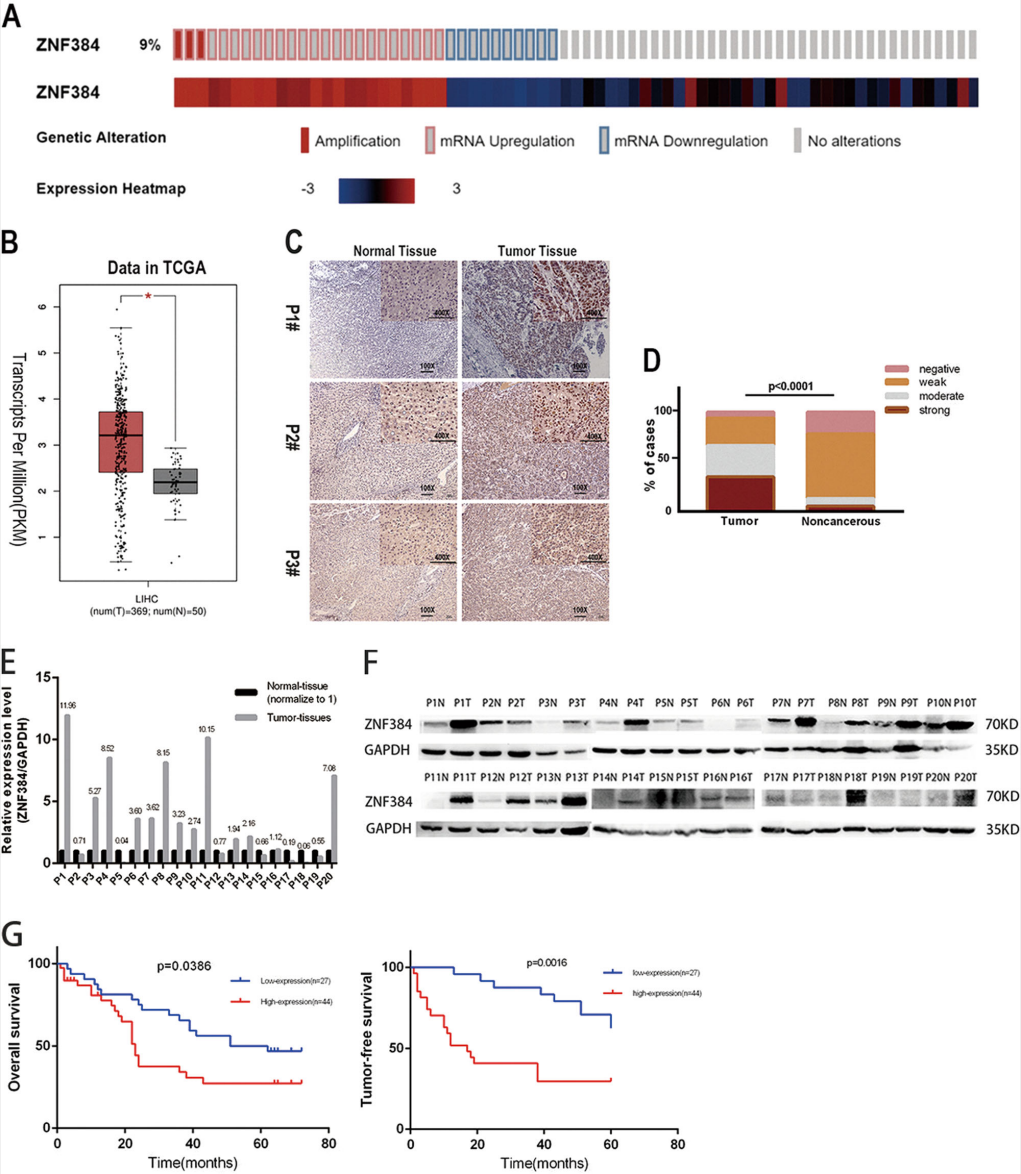
ZNF384 is frequently up-regulated in hepatocellular carcinoma (HCC) and associated with recurrence and poor prognosis of HCC patients. **a** The alter frequency and the expression changes of ZNF384 in HCC from TCGA database. **b** Upregulated ZNF384 mRNA expression in HCC compared with normal liver tissue in TCGA database. **c** Immunohistochemical staining of ZNF384 protein expression in 71 pairs of clinical HCC and adjacent noncancerous tissues. **d** Difference in IHC score of ZNF384 protein between HCC and adjacent noncancerous tissues. **e** Kaplan–Meier analysis showing that the expression level of ZNF384 in 71 HCC patient samples is significantly associated with poorer overall survival (log-rank test). **f** Kaplan–Meier analysis showing that the expression level of ZNF384 in 71 HCC patient samples is significantly associated with tumor-free survival (log-rank test)

To explore the potential role played by ZNF384 in the development of HCC, we obtained genetic information for ZNF384 in HCC from the TCGA database. We found that the rate of genetic alteration of ZNF384 was as high as 9.4% and that mRNA upregulation accounted for 70.6% of these alterations (Fig. 1a). We also found that ZNF384 expression was significantly increased in HCC tissues compared to that in adjacent normal liver tissues in the TCGA RNA-seq database (Fig. 1b). The protein expression of ZNF384 was further assessed in HCC samples and corresponding adjacent normal tissues (ANT) by IHC staining. These results showed that most HCC samples exhibited higher protein expression of ZNF384 than ANT (Fig. 1c, d). Figure 1e, f also show that most HCC samples had elevated ZNF384 protein expression.

### High expression of ZNF384 in HCC indicates a worse prognosis

The association of ZNF384 expression with overall survival was analyzed via Kaplan–Meier analysis in 71 patients with complete follow-up information. The high expression of ZNF384 predicted poorer prognosis in HCC patients (*P* = 0.0386, Fig. 1g). The high expression of ZNF384 was closely related to the recurrence of HCC (*P* = 0.0097). We also observed that high expression of ZNF384 always correlated with shorter tumor-free survival time in patients (*P* = 0.0016, Fig. 1g). The detailed patient information is shown in Table 1.

**Table 1:** Correlations between ZNF384 expression and clinicopathologic characteristics in 71 HCC patients

	Cases	ZNF384 expression		*P*-value
		Low (%)	High (%)	
Total	71	38.03	61.97	
*Age*				
<60	31	18.31	25.35	0.5228
≥60	40	19.72	36.62	
*Gender*				
Male	58	33.80	47.89	0.2250
Female	13	4.23	14.08	
*AFP (ng/ml)*				
<400	53	29.58	45.07	0.6406
≥400	18	8.45	16.90	
*HBsAg*				
Negative	17	7.04	16.90	0.4086
Positive	54	30.99	45.07	
*Size (cm)*				
<5	41	25.35	32.39	0.2393
≥5	30	12.68	29.58	
*Recurrence*				
No	31	23.94	19.72	0.0097
Yes	40	14.08	42.25	
*Liver cirrhosis*				
No	34	18.31	29.58	0.9730
Yes	37	19.72	32.39	

### Downregulated expression of ZNF384 inhibits HCC cells proliferation through G1/S phase transition arrest

**Fig. 2:**
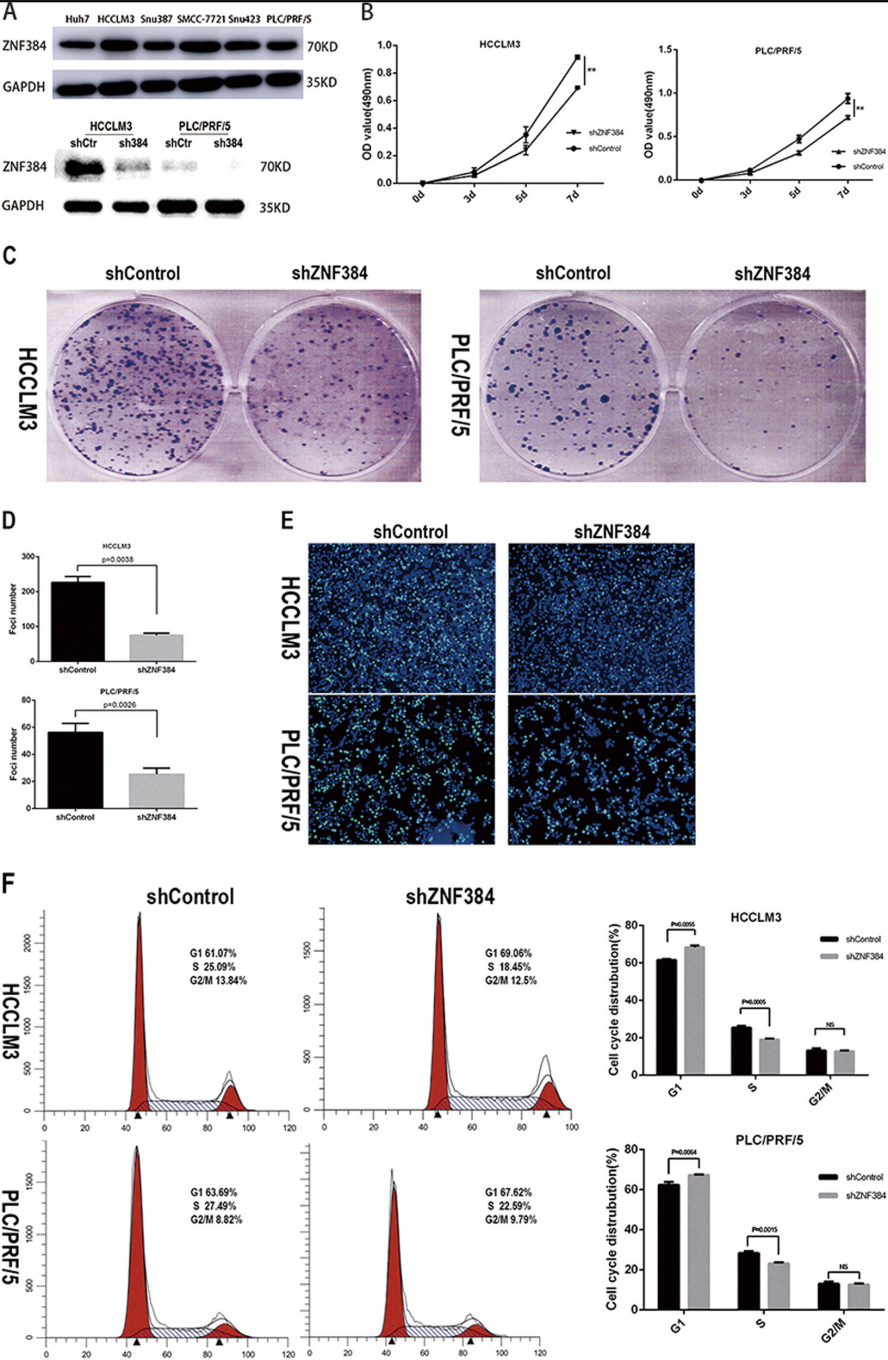
Knockdown of ZNF384 by shRNA inhibits the proliferation of hepatocellular carcinoma cells. **a** Western blot analysis of ZNF384 expression in HCCLM3 and PLC/PRF/5 cells treated with shRNA targeting ZNF384. Silencing ZNF384 in HCCLM3 and PLC/PRF/5 cells effectively inhibits cell growth (**b**) clone formation (**c**). Knockdown of ZNF384 in HCCLM3 and PLC/PRF/5 cell effectively inhibits G1/S phase transition in EdU immunofluorescence staining (**d**) and Flow cytometry analysis (**e**, **f**). All results are expressed as the mean ± SD of three independent experiments (*0.01 ≤ *P* < 0.05; **0.001 ≤ *P* < 0.01, ***P < 0.001)

To explore the role of ZNF384 in the development of HCC, we first verified the expression level of ZNF384 in liver cancer cell lines in order to select appropriate HCC cell lines. The results showed that ZNF384 was expressed at high levels in all HCC cell lines. Therefore, we selected HCCLM3, PLC/PRF/5, and Huh7 cells for the next experiment. We generated endogenous ZNF384 knockdown cell lines via shRNA in HCCLM3 and PLC/PRF/5 cells (Fig. 2a). MTS assays revealed that silencing ZNF384 in HCCLM3 and PLC/PRF/5 cells markedly inhibited cell growth (Fig. 2b). Similarly, ZNF384 knockdown reduced the colony formation ability of the HCCLM3 and PLC/PRF/5 cell lines (Fig. 2c, d). The results of the MTS and colony formation assays indicated that ZNF384 promoted cell growth. The effect of the ZNF384 protein on the cell cycle was analyzed via EdU immunofluorescence and flow cytometry, and the EdU immunofluorescence results showed that the proportion of cells in S phase was decreased when the expression of ZNF384 was silenced (Fig. 2e). In addition, the flow cytometric analysis results suggested that the cell cycle was arrested at the G1/S phase transition when ZNF384 expression was silenced (Fig. 2f).

**Fig. 3:**
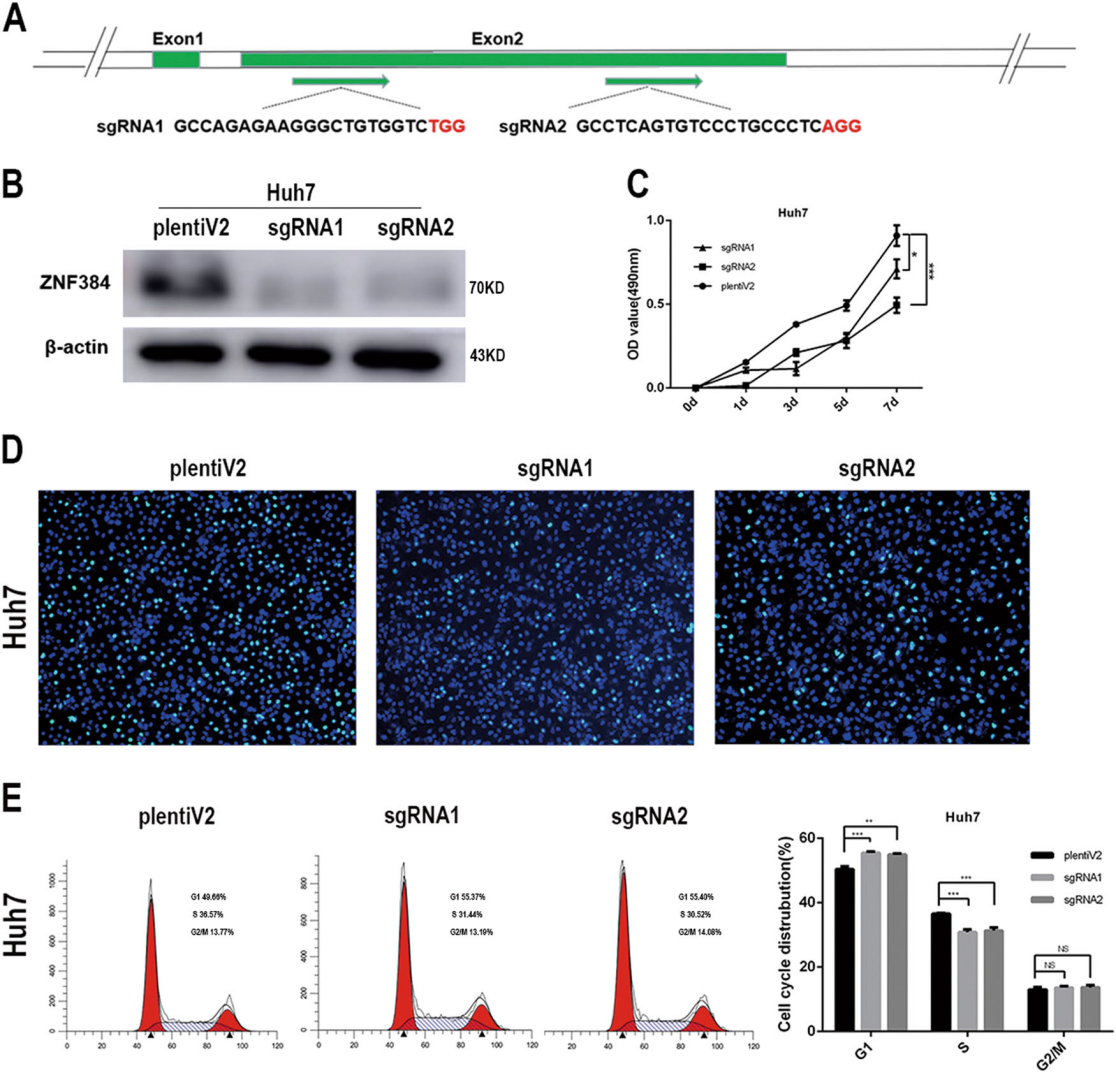
Verify the effect of ZNF384 on the proliferation ability of hepatocellular carcinoma cells by knocking-out ZNF384 through Crispr/CAS9. **a** The detail information of the two sgRNAs we chose. **b** Western blot analysis of ZNF384 expression in Huh7 cells treated with Crispr/CAS9 targeting ZNF384. **c** Knocking-out ZNF384 expression in Huh7 cells effectively inhibits cell growth. **e**, **f** Knocking-out ZNF384 expression in Huh7 cells effectively inhibits G1/S phase transition through Flow cytometry analysis. All results are expressed as the mean ± SD of three independent experiments (*0.01 ≤ *P* < 0.05; **0.001 ≤ *P* < 0.01, ***P < 0.001)

To verify that phenotypic changes in cell proliferation were indeed caused by the reduced expression of ZNF384 rather than by off-target effects, we designed single guide RNAs (sgRNAs) targeting the second exon of the ZNF384 gene (Fig. 3a) and generated a ZNF384 knockout Huh7 cell line via the CRISPR/Cas9 technique (Fig. 3b). The MTS assay results showed that cell proliferation was also slowed in the ZNF384-targeted knockout group compared with that in the control group (Fig. 3c). Similarly, we analyzed the impact of ZNF384 protein knockout on the cell cycle via EdU immunofluorescence and flow cytometry experiments; these results also indicated cell cycle arrest at the G1/S phase transition when ZNF384 was knocked out (Fig. 3d, e).

**Fig. 4:**
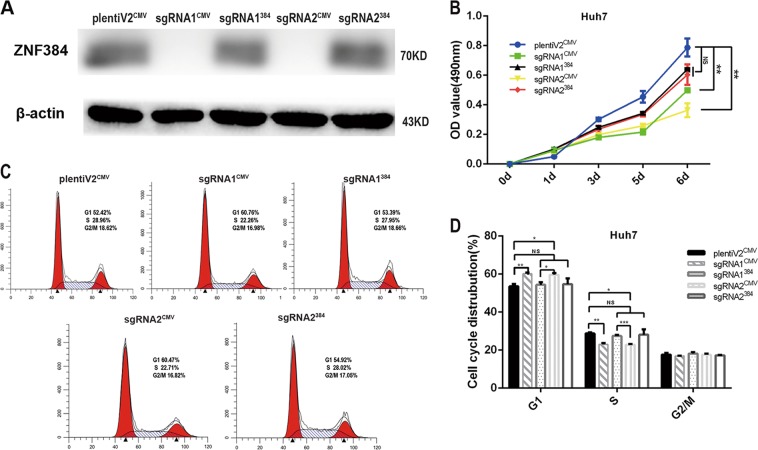
Restoring ZNF384 protein in sgRNA-transfected Huh7 cells. **a** Western blotting shows that the expression of ZNF384 protein restored in sgRNA-Huh7 cells. **b** Restoring ZNF384 protein could reinstate the ability of cell proliferation. **c**, **d** Restoring ZNF384 protein could eliminate the suppression of G1/S phase transition

Furthermore, we restored ZNF384 protein expression in ZNF384 knockout Huh7 cells (Fig. 4a). We found that the proliferative capacity of cells with restored ZNF384 expression was greater than that of knockout cells (Fig. 4b) and that the proportion of G1/S-phase cells could be restored to a level similar to that in the control groups (Fig. 4d).

### ZNF384 promotes tumor growth in vivo

**Fig. 5:**
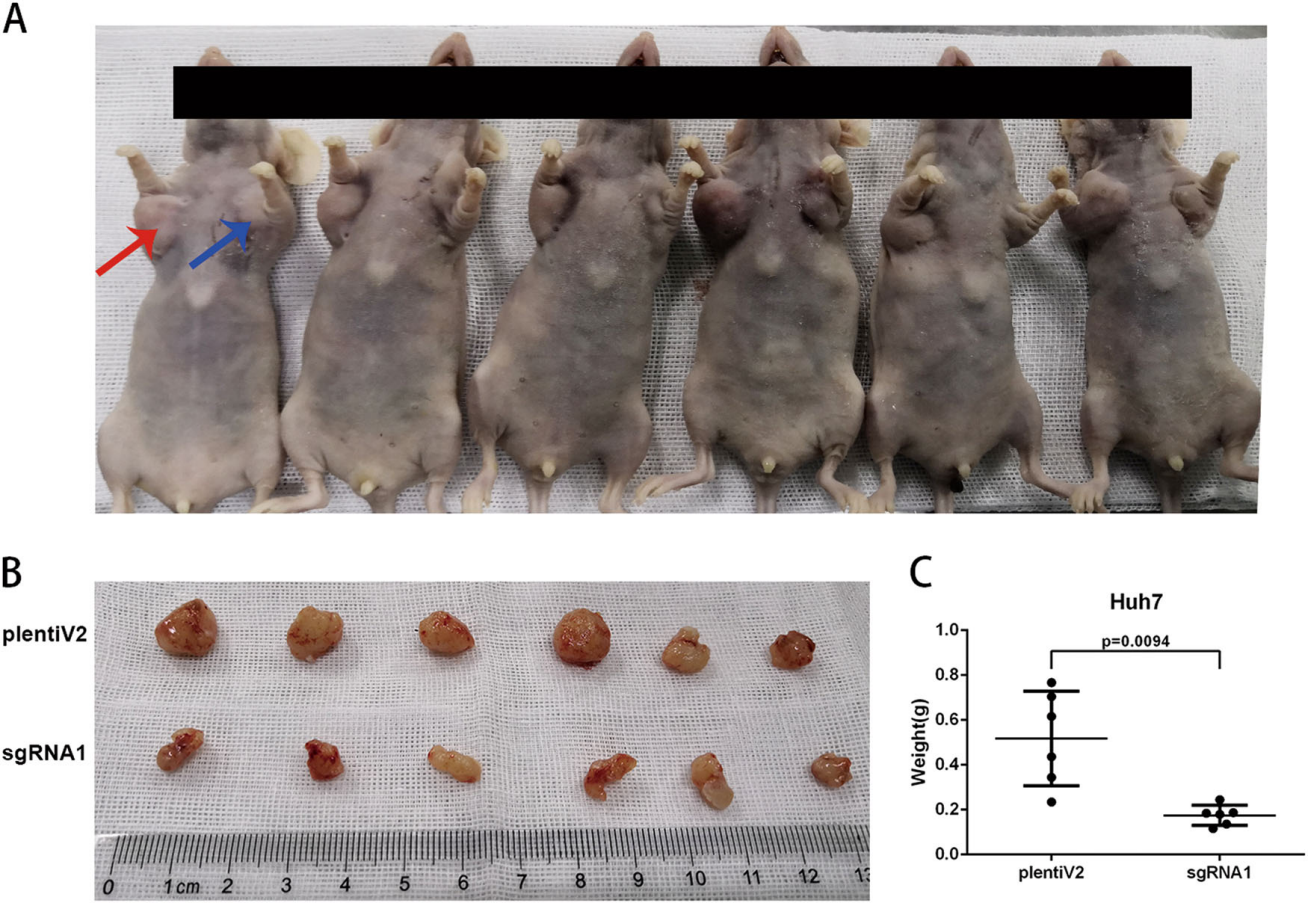
ZNF384 promoter tumor growth in vivo. **a** Images of xenograft tumors formed in nude mice injected with sgRNA1-Transfected Huh7 cells (Left, red arrows) and empty vector-transfected Huh7 cells (Right, blue arrows). **b** Tumors formed in nude mice. **c** Tumor weights were analyzed by independent Student’s *t*-test

An in vivo tumourigenesis assay was performed by subcutaneous injection of sgRNA1-Huh7 cells and control Huh7 cells into the left and right axillae of nude mice, respectively (Fig. 5a). Xenograft tumours were harvested after four weeks, and sgRNA1-Huh7 cells developed smaller tumours than control cells (*P* = 0.0094, Fig. 5b, c).

### ZNF384 influence the cell cycle by regulating the expression of Cyclin D1

**Fig. 6:**
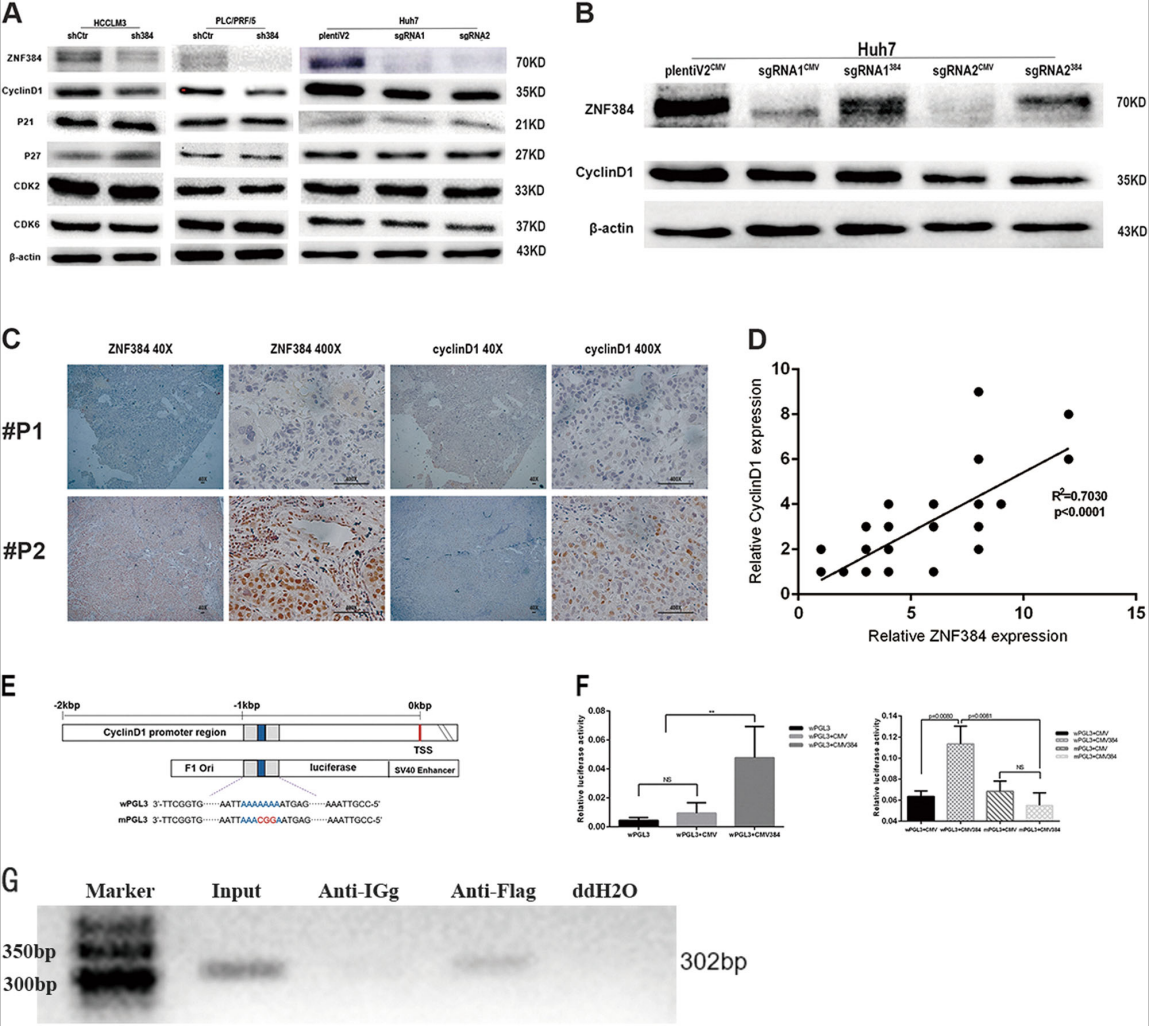
ZNF384 binds to the promoter region of cyclinD1 to regulate the expression of cyclin D1. **a** Western blotting shows that the expression of Cyclin D1 was down-regulated in both of shZNF384-transfected and sgRNA-transfected cells compared with empty vector-transfected cells. **b** Western blotting shows that the expression of Cyclin D1 recovered when we restored ZNF384 expression in sgRNA-transfected cells. **c** The expression of ZNF384 and Cyclin D1 in 60 HCC samples through IHC. **d** The correlation between the expression of ZNF384 and Cyclin D1 in correlation analysis (*n* = 60, part of dots was overlapped). **e** The structure of the luciferase plasmid in this study. **f** ZNF384 recognizes and bound to the promoter region of Cyclin D1 and regulates Cyclin D1 transcription. **g** Binding of ZNF384-Flag to the predicted combining capacity in Cyclin D1 promoter region by ChIP assays. Input DNA was used as positive control and ddH_2_O was used as a negative control

To elucidate the mechanism of G1-to-S phase arrest caused by ZNF384 protein downregulation, we examined the expression changes of proteins involved in the G1/S phase transition, including Cyclin D1, p21, p27, CDK2, and CDK6, by western blotting. The results showed that the expression of Cyclin D1 was significantly reduced in ZNF384 knockdown HCCLM3 and PLC/PRF/5 cells. The same results were observed in ZNF384 knockout Huh7 cells. When we restored ZNF384 expression in these knockdown and knockout cells, we found that the expression of Cyclin D1 was restored (Fig. 6b). Moreover, we detected a significant positive correlation between the expression of ZNF384 and cyclin D1 in 60 HCC samples (*P* < 0.0001, Fig. 6c, d).

### ZNF384 recognizes the promoter region of CCND1 and regulates its transcription

Previous results suggest a regulatory relationship between ZNF384 and Cyclin D1. Considering that ZNF384 is regarded to be a transcription factor, we speculated whether ZNF384 might directly regulate the transcription of Cyclin D1. Interestingly, we found through online analysis tools (http://jaspardev.genereg.net) that there might be ZNF384 recognition binding sites in the Cyclin D1 promoter region. Then, we used a luciferase reporter system and found that co-transfection of the ZNF384 plasmid and the wPGL3 plasmid caused a significant increase in luciferase units compared to that in the control groups (Fig. 6f). However, when we co-transfected the ZNF384 plasmid and mPGL3 plasmid, we did not observe significantly enhanced luciferase activity compared with that in the control groups (Fig. 6f). Thus, we performed ChIP analysis with cells overexpressing ZNF384-Flag. The results confirmed that ZNF384 is involved in the transcriptional regulation of Cyclin D1.

## Discussion

In recent years, with the development of gene sequencing technology, genomic analyses show promise for improving tumor characterization and helping us develop individual treatment strategies for HCC patients^14^. A better understanding of the genetic alterations in HCC could contribute to identifying potential driver mutations and discovering novel therapeutic targets in the future^15,16^. In this study, we showed that ZNF384, a gene with a genetic alteration rate of 9.4% in HCC, was overexpressed in HCC and that the expression level of ZNF384 was associated with the prognosis of patients. When we downregulated the expression of ZNF384 via shRNA or CRISPR/Cas9 in HCC cell lines, cell proliferation was slowed both in vitro and in vivo. Cell cycle arrest at the G1/S transition was observed in ZNF384 knockdown cells. Finally, we verified that ZNF384 binds to the promoter region of Cyclin D1 and regulates the expression of Cyclin D1.

ZNF384 is a highly conserved gene expressed in a variety of human tissues^17^. This pattern of widespread expression suggests that ZNF384 has essential, evolutionarily conserved biological functions. Given that HCC is a highly heterogeneous cancer, there are significant differences in the oncogene and tumor suppressor gene mutation maps and the gene expression profiles among liver cancer patients. It is worth noting that the tumor tissues of over 90% of the HCC patients in our study exhibited increased ZNF384 expression. It seems that ZNF384 plays a role as an oncogene in HCC. In a link to carcinogenesis, the recurrent rearrangement of ZNF384 with RNA-binding protein-coding genes such as EWSR1 or TAF15 have been observed and identified as oncogenic subtypes in acute leukemia (AL)^18^. Additionally, the transactivating properties of the fusion protein were found in NIH3T3 cells, which implied the oncogenic potential of ZNF384 as a fusion protein^19^. Furthermore, it is reported that overexpression of ZNF384 can promote metastasis in melanoma cells^12^. Therefore, it is reasonable to speculate that HCC cells increase their ability to survive by expressing high levels of ZNF384 through unknown mechanisms. More studies are required to explore the molecular details underlying the regulation of ZNF384 expression in HCC.

The cell cycle is a series of precisely regulated steps orchestrated by specific cyclins that act in association with cyclin-dependent kinases (CDKs)^20^. In normal cells, the cell cycle is precisely regulated. However, the ability to sustain unscheduled proliferation is a notable hallmark of cancer^21^. In this study, we found that reduced expression of ZNF384 blocked HCC cells from progressing from G1 phase to S phase with the downregulation of Cyclin D1 expression. Cyclin D1 is a protein encoded by the CCND1 gene, and it belongs to the highly conserved cyclin family, whose members are characterized by periodic changes in protein expression throughout the cell cycle^22^. The function of cyclin D1 in cell cycle control is believed to be mediated through interaction with CDK4 and/or CDK6 to promote G1/S phase transition^23,24^. Therefore, overexpression of Cyclin D1 is frequently observed to promote cancer cell proliferation in a variety of cancers, including HCC. The relationship between ZNF384 and Cyclin D1 was further confirmed in clinical samples by IHC. In addition, it is reported that advanced-stage HCCs potentially harbor more Cyclin D1 amplifications, and the amplification of Cyclin D1 is also associated with poor prognosis in HCC^25^. This finding is consistent with our finding in this study that high ZNF384 expression often predicts a worse prognosis for patients.

ZNF384 is a transcription factor containing a C2H2 zinc finger structure, and previous studies have shown that it regulates MMP1, MMP3, and COL1A1 gene transcription^26–28^. It is predicted that the transcriptional recognition site is a sequence with adenine enrichment. Moreover, we found an adenine-enriched sequence in the promoter region of Cyclin D1. Thus, we postulated that Cyclin D1 might also be a target gene of ZNF384, a hypothesis that was verified through a luciferase assay. These results demonstrate that ZNF384 specifically targets the promoter region of Cyclin D1 and regulates the expression of Cyclin D1.

In summary, our study first described the role of ZNF384 as an oncogene in the development of HCC. ZNF384 promotes the proliferation of HCC cells by upregulating the expression of Cyclin D1. In addition, we provide evidence that ZNF384 directly regulates Cyclin D1 transcription in HCC. In the future, ZNF384 might serve as a prognostic or predictive factor and as a potential therapeutic target in HCC diagnosis and treatment.

## Materials and methods

### Patients and specimens

We collected 51 paired HCC samples and adjacent noncancerous tissues from patients who underwent liver resection in Sir Run Run Shaw Hospital (SRRSH) from January 2006 to December 2010 and obtained another 20 paired HCC samples and adjacent noncancerous tissues from patients who underwent liver resection in Sir Run Run Shaw Hospital (SRRSH) from May 2017 to September 2017. All specimens were immobilized in formalin immediately and further embedded for subsequent pathological testing. In addition, we also acquired 20 pairs of fresh frozen tissue specimens from the hospital tissue bank for quantitative polymerase chain reaction (PCR) analysis and western blot analysis. Detailed information about the clinical and pathological characteristics is listed in Table 1. The American Joint Committee on Cancer (AJCC) staging system was used to evaluate tumor stages, and the Barcelona Clinic Liver Cancer (BCLC) staging system was used to define clinical stages. The study was approved by the Medical Ethics Committee of SRRSH, and all patients were informed.

### Quantitative real-time PCR analysis

Total RNA was extracted from tissue samples using Trizol reagent (Thermo Fisher Scientific cat#204211) according to the manufacturer’s instructions. cDNA was synthesized from 1 μg of RNA of each sample using an iScript cDNA synthesis kit (YEASEN, cat#11121ES60). qRT-PCR was performed using a qPCR SYBR Green Master Mix (YEASEN, cat#11198ES08). The ZNF384 primer sequences were as follows: forward, 5′-GTCTCAGGTCAGATCGAGAACA-3′ and reverse 5′-ACTCTGTGTCCATACTGATGCC-3′. The GAPDH primer sequences were as follows: forward, 5′-GTGAAGCAGGCGTCGGA′ and reverse, 5′-AGCCCCAGCGTCAAAGG-3′. Each sample was tested in triplicate. Data were analyzed using the 2^−ΔΔCT^ calculation method.

### Cell culture

The HCC cell lines Huh7, HCCLM3, Snu423, Snu387, SMCC-7721, and PLC/PRF/5 were kindly provided by the Cang laboratory at Zhejiang University. Huh7, HCCLM3, and PLC/PRF/5 cells were cultured in DMEM (Gibco, cat#C11995500BT) supplemented with 10% fetal bovine serum (FBS) (Cellmax, cat#SA102.02). Snu423, Snu387, and SMCC-7721 cells were cultured in RPMI-1640 (Gibco, cat# C11975500BT) supplemented with 10% FBS (Cellmax, cat#SA102.02). The culture environment was maintained at 37 °C with 5% CO_2_.

### Plasmid constructs

First, we obtained the sequence for shRNA targeting ZNF384 from http://www.sigmaaldrich.com. The shRNA sequence was as follows: forward, 5′-CCGGCCCGAGATGAATGACCCTTATTTCAAGAGAATAAGGGTCATTCATCTCGGGTTTTTTGGTACC-3′ and reverse, 5′-AATTGGTACCAAAAAACCCGAGATGAATGACCCTTATAAGTTCTCTATAAGGGTCATTCATCTCGGG-3′. Then, we cloned the shRNA into the pLKO.1 puro plasmid (Sigma, cat#SHC001) at the AgeI and EcoRI restriction sites according to the manufacturer’s instructions. We also designed the single guide RNA (sgRNA) using the online sgRNA design website (http://crispr.mit.edu). The sgRNA was cloned into the lentiCRISPRv2 plasmid (Addgene, cat#52961) according to the established protocol. Full-length ZNF384 cDNA was generously provided by the Jiahuai Han laboratory (Xiamen University). The cDNA was amplified and cloned into the p3xFlag-CMV-7.1 expression vector (Sigma, cat#E4026) using XbaI and BamHI. Finally, we amplified the fragment located at the promoter region of Cyclin D1. Subsequently, this fragment was PCR amplified and cloned upstream of the firefly luciferase gene in the pGL3-Enhancer vector (Promega, cat#E1771) using NheI and HindIII. This plasmid was termed wPGL3. Using a Mut Express Multis Fast Mutagenesis Kit (Vazyme, cat#C215-01), we performed site-directed mutagenesis of the ZNF384 binding sites in the Cyclin D1 promoter region. This plasmid was termed mPGL3. Detailed information for the primers we used is provided in Table 2.

**Table 2:** Detail information for the primers we used. F, forward; R: reverse

Name	Sequences (5′-3′)
sgRNA-1	F: CACCGGCCTCAGTGTCCCTGCCCTC
sgRNA-1	R: AAACGAGGGCAGGGACACTGAGGCC
sgRNA-2	F: CACCGGCCAGAGAAGGGCTGTGGTC
sgRNA-2	R: AAACGACCACAGCCCTTCTCTGGCC
XbaI-ZNF384	F: GCCTCTAGAATGGAAGAATCTCACTTCA
BamHI-ZNF384	R: TTTGGATCCCTAAGAGCTGGCCAGGTGC
NheI-CCND1	F: CTAGCTAGCCACCCCCAACAAAACCAATT
HindIII-CCND1	R: CCCAAGCTTTTCCTACCTTGACCAGTCGG
Mutate-CCND1	F: ATTTCTTTTTTAATTAAACGGAATGAGTCAG
Mutate-CCND1	R: GATCTCCATTCTGACTCATTCCGTTTAATTA

### Lentivirus production and transduction

Lentivirus expressing shRNA was produced by co-transfecting HEK293T cells with a mixture of shZNF384 or shControl plasmid, the Gag-Pol packaging plasmid psPAX2 (Addgene, cat#12260), and the envelope plasmid pMD2G (Addgene, cat#12259) at a 4:3:2 ratio using Lipofectamine 2000 transfection (lip2000) reagent (Thermo Fisher, cat#11668019). Lentivirus expressing sgRNA and cas9 was produced by co-transfecting HEK293T cells with a cocktail of gRNA/Cas9-expressing lentiCRISPRv2, Gag-Pol packaging plasmids, including psPAX2 (Addgene, cat#12260), and B19/VSVG (Addgene, cat#88865) at a ratio of 3:4:1 using lip2000 reagent. Virus particles were harvested 48 h after transfection. Lentivirus-infected cells were screened with the corresponding concentration of puromycin (Thermo Fisher, cat#A1113802: Huh7, 1 µg/µl; PLC/PRF/5, 1 µg/µl; HCCLM3, 10 µg/µl).

### MTS cell proliferation assay and colony formation assay

For the cell growth assay, we seeded 1 × 10^3^ cells per well into 96-well plates. After the cells were attached, we added 5 mg/ml MTS (Promega, cat#G3580) to each well at 0 h for 1 day, 3 days, 5 days, and 7 days, and the absorbance values were measured at a wavelength of 490 nm by spectrophotometry.

In the colony formation assay, 1 × 10^3^ cells were seeded in 6-well culture plates. Two weeks later, the cells were fixed in 4% paraformaldehyde, stained with crystal violet solution (Beyotime Biotechnology, cat#C0121) and counted under a microscope.

### EdU incorporation assay

ZNF384 knockdown or knockout HCC cells and their corresponding control cells were seeded in a 24-well culture plate at 2 × 10^5^ cells/well and incubated for 24 h. A 5-ethynyl-20-deoxyuridine (EdU) assay using an EdU assay kit (Beyotime Biotechnology, cat#C0071S) was used to assess cell proliferation according to the manufacturer’s protocol. First, cells were incubated with 10 μM EdU for 3 h at 37 °C and were then fixed with 4% paraformaldehyde. After the samples were treated with 0.3% Triton X-100 for 10 min, the cells were stained with Azide 488 for 30 min. Subsequently, cell nuclei were stained with Hoechst 33342 for 10 min. Samples were analyzed by a fluorescence microscope (Olympus).

### Flow cytometry analysis

Cell cycle distribution was analyzed by flow cytometry (BD LSRFortessa^TM^ X-20). Cells were treated with cell cycle staining buffer (MultiSciences, cat#CCS01) according to the manufacturer’s instructions.

### Western blotting and antibodies

Cells were harvested and lysed in radioimmunoprecipitation assay buffer (1% NP40, 0.5% sodium deoxycholate, 0.1% sodium dodecyl sulfate, 0.03% aprotinin, 10 ng/ml phenylmethylsulfonyl fluoride, and 1 μM sodium orthovanadate) at 4 °C for 30 min. The protein concentration was quantified using a BCA protein assay kit (Thermo Fisher Scientific, cat#A53227). Proteins were separated via 10% SDS-polyacrylamide gel electrophoresis (SDS-PAGE). Next, samples were transferred to 0.22 μm-thick polyvinylidene difluoride (PVDF) membranes (Merck Millipore, Billerica, MA, USA). Non-specific binding sites on the membranes were blocked for 1 h with 5% non-fat milk (BD, cat#232100). After blocking, membranes were incubated first with a primary antibody and then with a secondary antibody. Finally, immunoreactions were visualized using Clarity Western ECL substrate (Bio-Rad, cat#VL001) and the blots were imaged using a luminescent image analyser (Fujifilm, Tokyo, Japan). The following antibodies were used: anti-ZNF384 (Atlas Antibodies, HPA004051), anti-Cdk2 (Abcam, cat#ab32147), anti-Cdk6 (Abcam, cat#ab124821), anti-Cyclin D1 (Abcam, cat#ab134175), anti-p21 (Abcam, cat#ab109199), anti-p27 (Abcam, cat#ab32034), anti-DDDDK (Abcam, cat#ab1162), and anti-GAPDH (Abcam, cat#ab181602).

### Immunohistochemical staining (IHC)

IHC was used to evaluate the expression of ZNF384 in HCC tissues and paired noncancerous tissues. Paraffin-embedded sections were provided by the department of pathology. First, these sections were deparaffinized and rehydrated. For antigen retrieval, the sections were immersed in 10 mM citrate buffer (pH 6.0) and boiled for 10 min in a microwave oven. Then, endogenous peroxidase activity was blocked with 3% hydrogen peroxide for 10 min. Non-specific binding sites were blocked with 5% normal goat serum for 30 min. The sections were incubated with an antibody against ZNF384 (1:100, Atlas Antibodies, cat#HPA004051) overnight at 4 °C. The sections were then incubated with the secondary antibody, and the expression of ZNF384 in the tissues was observed via microscopy after DAB staining and haematoxylin staining. The score was evaluated by two pathologists blinded to the clinicopathological data using a German immunoreactivity score^29^.

### In vivo tumorigenicity assay

A xenograft mouse model was used to verify the oncogenic function of ZNF384 in vivo. BALB/c nude male mice (4 weeks of age) were subcutaneously injected with Huh7/sgRNA1 knockout cells (1 × 10^6^, subcutaneous) or Huh7/lentiV2 control cells (1 × 10^6^, subcutaneous) in 150 μl of PBS. Mice were sacrificed after four weeks, and the tumours were harvested and measured. The BALB/c nude mice (*n* = 6, male, 4 weeks of age) were obtained from Shanghai Laboratory Animal Center and housed in the laboratory animal research center of SRRSH. All animal experiments were conducted with consent from the Committee of the Use of Live Animals in Teaching and Research at Sir Run Run Shaw Hospital.

### Dual-luciferase reporter assay

HEK293T cells were separately transfected with the vectors wPGL3 or mPGL3. ZNF384 was overexpressed by the addition of the ZNF384 expression vector (pMSCV-Flag-hSOX2, Addgene, cat#2007). Luciferase activity was normalized to that of cells co-transfected with the pRL Renilla expression vector (Promega, cat#E2231). Lysates were analyzed with a dual luciferase kit (Promega, cat#E1980).

### Chromatin immunoprecipitation assay

LM3 cells were transfected with the CMV-Flag plasmid for 3 days and cross-linked with 1% formaldehyde for 10 min at 37 °C in 10 ml of DMEM. Then, we added 1.1 ml of glycine solution (10×) to the reactions at room temperature. After 5 min, cells were washed three times with PBS containing 1 mM PMSF and resuspended in 200 µl of lysis buffer containing 1 mM PMSF. Subsequently, cell lysates were treated with a Misonix Sonicator 3000 Homogenizer (Mandel Scientific Company Inc., Guelph, ON, Canada) to fragment genomic DNA into sizes of 200–800 bp. After the samples were centrifuged at 4 °C and 12,000 × *g* for 5 min, we removed the supernatant and added 1.8 ml of ChIP Dilution Buffer containing 1 mM PMSF to the supernatant. Twenty microliters of each sample were removed and used as input for subsequent detection. The remaining sample volumes were precleared with 70 μl of protein A/G agarose for 30 min at 4 °C. After removing the protein A/G agarose, anti-DDDK flag and anti-IgG antibodies were used to immunoprecipitate the samples on a shaker at 4 °C overnight. After centrifugation, the supernatant was discarded, and the protein A/G agarose was washed successively in Low Salt Immune Complex Wash Buffer, High Salt Immune Complex Wash Buffer, LiCl Immune Complex Wash Buffer and TE Buffer. Finally, DNA was eluted from the protein A/G agarose with elution buffer, and 20 µl of 5 M NaCl was added to the solution to eliminate cross-linking between proteins and genomic DNA. The DNA was analyzed via PCR with the following primers: forward primer, 5′-tctaaaggtgaagggacgtc-3′ and reverse primer, 5′-ccttcatcttgtccttctagc-3′.

### Statistical analysis

All results are presented as the means ± SDs. The differences between categorical variables were assessed by the *χ*^2^ test, and the differences between two groups were assessed by Student’s *t*-test. HCC survival data such as OS and DFS were analyzed with Kaplan–Meier curves and the log-rank test. Statistical analysis was performed using SPSS 22.0 software (SPSS Inc., Chicago, IL, USA). A *P*-value of < 0.05 was considered statistically significant.
